# Randomised trial of once-daily vilanterol in children with asthma on inhaled corticosteroid therapy

**DOI:** 10.1186/s12931-016-0353-4

**Published:** 2016-04-05

**Authors:** Amanda J. Oliver, Ronina A. Covar, Caroline H. Goldfrad, Ryan M. Klein, Søren E. Pedersen, Christine A. Sorkness, Susan A. Tomkins, César Villarán, Jonathan Grigg

**Affiliations:** GlaxoSmithKline, Stockley Park West, 1 − 3 Iron Bridge Road, Uxbridge, Middlesex UB11 1BT UK; Department of Pediatrics, National Jewish Health, Denver, CO USA; Southern California Clinical Trials, Newport Beach, CA USA; University of Southern Denmark, Pediatric Research Unit, Kolding Hospital, Kolding, Denmark; University of Wisconsin School of Medicine and Public Health, Madison, WI USA; Clinica Ricardo Palma, Javier Prado Este 1166 San Isidro, Lima, Perú; Blizard Institute, Queen Mary University London, London, UK

**Keywords:** Asthma, Children, Dose response, Efficacy, Fluticasone propionate, Safety, Vilanterol

## Abstract

**Background:**

Inhaled corticosteroids (ICS) are effective maintenance treatments for childhood asthma; however, many children remain uncontrolled. Vilanterol (VI) is an inhaled long-acting beta-2 agonist which, in combination with the ICS fluticasone furoate, is being explored as a once-daily treatment for asthma in children. We evaluated the dose–response, efficacy, and safety of once-daily VI (6.25 μg, 12.5 μg and 25 μg) administered in the evening over 4 weeks, on background fluticasone propionate (FP) in children with asthma inadequately controlled on ICS.

**Methods:**

This was a Phase IIb, multicentre, randomised, double-blind, parallel-group, placebo-controlled study in children ages 5–11 years with persistent asthma on ICS and as-needed short-acting beta-agonist. The study comprised a 4-week run-in, 4-week treatment period, and 1-week follow-up. From study start, children replaced their current ICS with open-label FP 100 μg twice daily. Children were randomised to receive placebo, VI 6.25 μg, VI 12.5 μg or VI 25 μg once daily. Primary endpoint was treatment difference between VI 25 and placebo groups in mean change from baseline in evening peak expiratory flow averaged over the 4-week treatment. Secondary endpoints included change from baseline in trough forced expiratory volume in one second (FEV_1_) at Week 4 and change from baseline in percentage of rescue-free and symptom-free 24-h periods. Safety assessments included incidence of adverse events (AEs) and asthma exacerbations.

**Results:**

In total, 456 children comprised the intention-to-treat population. The adjusted treatment difference between VI 25 and placebo groups for the primary endpoint was not statistically significant (*p* = 0.227) so no statistical inference was made for other VI dose comparisons or other endpoints. No difference in change from baseline in trough FEV_1_ was observed for any VI treatments versus placebo; however, VI 25 resulted in an additional 0.6 rescue-free days and 0.7 symptom-free days per week versus placebo. The incidence of AEs was slightly higher in the VI groups (28–33 %) versus placebo (22 %). Nine children experienced asthma exacerbations during the treatment period.

**Conclusion:**

VI plus FP did not result in significant improvements in lung function versus placebo plus FP, but was well tolerated at all doses assessed.

**Trial registration:**

NCT01573767 (ClinicalTrials.gov).

## Background

Asthma is common in children and is a leading cause of childhood hospitalisation [1]. National and international guidelines advocate the use of inhaled long-acting beta-2 agonists (LABA) in combination with inhaled corticosteroids (ICS) as maintenance therapy for children ages 5–11 years who remain symptomatic despite medium doses of ICS [2, 3]. A network meta-analysis including over 12,000 patients across 35 studies found that combined ICS/LABA treatments were more effective than low-dose ICS in preventing asthma exacerbations among paediatric patients [4]. In addition, studies indicate that the addition of LABA to a low dose of ICS can provide benefits such as improved asthma control in some children compared with increasing the ICS dose [5] and a reduced impact on growth by 1.2 cm/year compared with double the dose of ICS [6].

Despite the availability of effective therapies, many children with asthma remain uncontrolled, with low adherence proposed as a potential contributing factor [7, 8]. In addition, studies in adult and adolescent patients using dry powder inhalers for treatment of asthma and chronic obstructive pulmonary disease have shown that adherence with a once-daily regimen is greater than with a twice-daily regimen [9, 10]. However, currently available ICS/LABA combination therapies require a twice-daily regimen [11, 12].

Vilanterol (VI) is a potent, inhaled LABA which, in combination with the ICS fluticasone furoate (FF), has been approved for the treatment of asthma in adults and adolescents in the EU, and in adults in the US [13–15]. In preclinical in vitro studies, VI demonstrated a selectivity profile for beta-2 adrenoceptors (over beta-1 and beta-3 adrenoceptors) that was similar to salmeterol and greater than formoterol and indacaterol [16]. Persistence and reassertion studies also showed a persistence of action comparable with indacaterol and greater than formoterol [16]. VI is currently being explored as the LABA component of FF/VI for once-daily treatment for asthma in children [17]. The objective of this study was to evaluate the dose–response, efficacy, and safety of three doses of VI (6.25 μg, 12.5 μg and 25 μg) administered once daily in the evening over a 4-week treatment period whilst on background fluticasone propionate (FP) therapy to children with asthma who were inadequately controlled on ICS.

## Methods

### Study design

This was a Phase IIb, multicentre, randomised, double-blind, parallel-group, placebo-controlled (with rescue medication) study in children ages 5–11 years with persistent asthma who were still symptomatic on low-dose ICS (ClinicalTrials.gov identifier NCT01573767). The study design consisted of a 4-week run-in period, a 4-week treatment period and a 1-week follow-up (contact) period. At the start of the run-in period, children replaced their current asthma medication with open-label FP 100 μg twice daily via DISKUS®/ACCUHALER® (GlaxoSmithKline), which they continued to take for the duration of the study. A total of 73 centres in 14 countries (Argentina, Chile, Georgia, Germany, Japan, Mexico, Peru, Philippines, Poland, Puerto Rico, Slovakia, South Africa, United States of America and Ukraine) randomised subjects.

### Participants

Children eligible for inclusion were males and pre-menarchial females with inadequately controlled asthma, ages 5–11 years, with at least a 6-month history of asthma and who had been receiving a stable dose of a ICS (total daily dose of FP 200–250 μg or equivalent) and rescue short-acting inhaled beta-agonist (SABA) for at least 4 weeks prior to screening. Eligible children had a pre-bronchodilator peak expiratory flow (PEF) of 50−90 % of their best post-bronchodilator value. Excluded children had: a history of life-threatening asthma; a change in asthma medication within 4 weeks of screening; an asthma exacerbation (defined as either requiring the use of systemic corticosteroids for ≥3 days, a depot corticosteroid injection within 3 months prior to screening, or hospitalisation for asthma within 6 months prior to screening); or a concurrent respiratory disease or any other clinically significant medical condition.

At the end of the run-in period, children eligible for randomisation had a pre-bronchodilator PEF of 50−90 % of their best post-bronchodilator value, had demonstrated symptoms of asthma and/or daily use of albuterol/salbutamol on ≥3 of the last 7 consecutive days of the run-in period, complied with the run-in medication on ≥4 of the last 7 consecutive days of the run-in period and had completed all questions on the Daily Diary on ≥4 out of the 7 days during the screening period. Children could not have experienced an asthma exacerbation between screening and randomisation.

The study was conducted in accordance with the ethical principles outlined in the Declaration of Helsinki and was approved by the relevant ethics committee or institutional review board at each investigational centre. Written informed consent was obtained from two parents/legal guardians. If applicable, the child had to be able and willing to give assent to take part in the study according to the local requirements. The investigator was accountable for determining a child’s capacity to assent.

### Treatment and assessments

Children were randomly assigned (1:1:1:1) to receive either placebo, VI 6.25 μg, VI 12.5 μg, or VI 25 μg once daily as their double-blind evening treatment (between 3 pm and 9 pm) in addition to continuing open-label FP 100 μg twice daily. Randomisation was performed via an interactive voice response system whereby children were assigned a randomisation number from a randomisation schedule created by GSK. Children were issued with a daily eDiary and a peak flow meter, and recorded morning and evening PEF, daytime and night time asthma symptom scores, day and night inhalations of SABA and the use of open-label ICS medication during the run-in period. Pre-dose forced expiratory volume in one second (FEV_1_) was measured electronically by spirometer at evening study visits at screening, randomisation, Week 2 and Week 4. These measurements were taken within ±1 h of the time FEV_1_ was measured at baseline and approximately 24 h after the child’s last evening dose. Children who were unable to perform acceptable quality FEV_1_ at randomisation did not have to perform the spirometry assessments at subsequent study visits, however, data from these children were still collected for all other endpoints.

The primary endpoint was the mean change from baseline in daily pre-dose trough evening PEF, recorded using an eDiary, averaged over the 4-week treatment period. Secondary endpoints were: 1) change from baseline in evening study visit trough FEV_1_ at the end of the 4-week treatment period (using last observation carried forward [LOCF] for imputation of missing post-baseline FEV_1_ values); 2) change from baseline in percentage of rescue-free and symptom-free 24-h periods averaged over the 4-week treatment period; 3) change from baseline in morning PEF averaged over the 4-week treatment period; and 4) mean change from baseline in morning and evening PEF over the last 7 days of the treatment period. Other endpoints were change from baseline in childhood asthma control test (cACT) score at the end of the 4-week treatment period and the percentage of subjects controlled, defined as a cACT score ≥20. A post-hoc analysis was carried out to determine the change from baseline in cACT score according to whether children had a baseline cACT score of <20 or ≥20.

Pharmacokinetic (PK) blood samples were collected pre-dose and 10−15 min post-dose during the Week 4 visit. Plasma samples were analysed for VI using techniques based on solid phase extraction followed by high-performance liquid chromatography and tandem mass spectrometry analysis (the lower limit of quantification [LLQ] was 10 pg/mL, using a 200 μL aliquot).

Safety was assessed by incidence of adverse events (AEs) and exacerbations throughout the 4-week treatment period; vital signs (pulse rate, systolic and diastolic blood pressure [BP]) at randomisation and at Week 2, Week 4 or early withdrawal; pre-dose and post-dose electrocardiogram (ECG) at randomisation and at Week 4 or early withdrawal; and laboratory assessments (haematology, clinical chemistry and liver function tests) at screening, Week 4 or early withdrawal.

### Statistical analysis

With 460 children (115 per treatment group) the study had 90 % power assuming a difference of 12 L/min between VI and placebo in evening PEF, a standard deviation of 28 L/min and significance declared at the two-sided 5 % level. In order to account for multiplicity across treatment comparisons for the primary efficacy endpoint, a step-down closed testing procedure was applied whereby inference for VI 12.5 μg versus placebo was dependent upon statistical significance having first been achieved for VI 25 μg versus placebo. Similarly, inference for VI 6.25 μg versus placebo was dependent upon statistical significance having first been achieved for VI 12.5 μg versus placebo.

The intention-to-treat (ITT) population comprised all children randomised to treatment who received ≥1 study medication; the per-protocol (PP) population comprised all children in the ITT population who did not have any full protocol deviations; and the PK population consisted of children in the ITT population for whom a PK sample was obtained and analysed for VI.

The analysis of the primary efficacy endpoint was performed using an analysis of covariance (ANCOVA) model allowing for the effects due to baseline evening PEF, region, sex, age and treatment group on the ITT and PP populations. Missing data were not imputed in this analysis. Five sensitivity analyses were performed to examine the impact of missing data on the primary endpoint: one mixed modelling repeated measures (MMRM) model analysis, and four multiple imputation sensitivity analyses. The MMRM analysis allowed for effects due to baseline evening PEF, region, sex, age, week and treatment group, including week-by-treatment and week-by-baseline interaction terms. The four imputation sensitivity analyses analysed the mean change from baseline in evening PEF using a Missing at Random approach, a Copy Increment from Reference approach, a Jump to Reference approach and a Copy Reference approach. An average treatment effect across Weeks 1−4 was obtained for all sensitivity analyses of the primary endpoint. Three further supporting analyses were also performed on the primary endpoint; these were dose response models, Bayesian analyses with non-informative priors and a MMRM model presenting estimates from each week.

Statistical analyses for secondary endpoints were performed using ANCOVA models with effects due to baseline, region, sex, age and treatment group. For trough FEV_1_ and morning/evening PEF at the endpoint, missing data was imputed using LOCF. Programming was performed using SAS Version 9.1.3 or later.

Percent predicted FEV_1_ values at screening and baseline were not pre-specified and are reported as posthoc analyses.

## Results

### Demographics

**Fig. 1:**
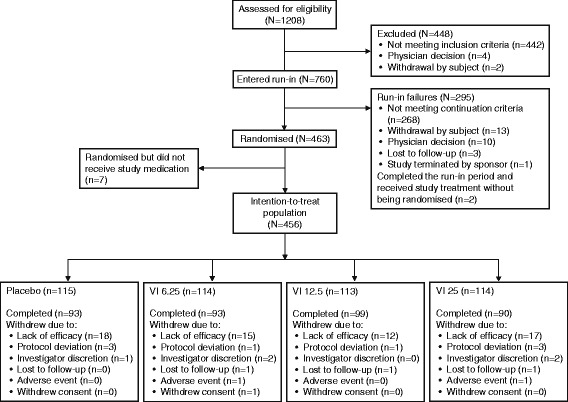
Participant flow diagram. Abbreviations: *VI*, vilanterol

A total of 1208 children were screened; 463 children were randomised with 456 (98 %) children included in the ITT population (Fig. 1). Overall, 81 (18 %) children withdrew prematurely from this 4-week study, the majority (62 children, 14 %) withdrawing due to lack of efficacy. The study period, from the first screening to the last visit, was from April 2012 to April 2014. Demographics were comparable between the four treatment groups and are summarised in Table 1. Lung function measures at screening and baseline are shown in Table 2. PEF and FEV_1_ values were similar across treatment groups and stable during the run-in period with open-label FP.

**Table 1:** Summary of demographic characteristics and baseline characteristics (ITT population)

Demographic	Placebo	VI 6.25 μg	VI 12.5 μg	VI 25 μg	Total
	*N* = 115	*N* = 114	*N* = 113	*N* = 114	*N* = 456
Sex, *n* (%)					
Female	50 (43)	43 (38)	42 (37)	45 (39)	180 (39)
Male	65 (57)	71 (62)	71 (63)	69 (61)	276 (61)
Age, years					
Mean (SD)	8.0 (1.81)	8.0 (1.95)	7.9 (1.74)	7.9 (1.72)	7.9 (1.80)
Age group, *n* (%)					
5–7 years	51 (44)	48 (42)	42 (37)	46 (40)	187 (41)
8–11 years	64 (56)	66 (58)	71 (63)	68 (60)	269 (59)
Race, *n* (%)					
White	68 (59)	63 (55)	55 (49)	62 (54)	248 (54)
White and American Indian or Alaska Native	20 (17)	19 (17)	26 (23)	24 (21)	89 (20)
American Indian or Alaska Native	16 (14)	21 (18)	17 (15)	18 (16)	72 (16)
Asian	6 (5)	6 (5)	7 (6)	6 (5)	25 (5)
African American/African heritage	5 (4)	5 (4)	5 (4)	3 (3)	18 (4)
White and African American/African heritage	0	0	2 (2)	0	2 (<1)
African American/African heritage and American Indian or Alaska Native	0	0	1 (<1)	0	1 (<1)
African American/African heritage and Asian	0	0	0	1 (<1)	1 (<1)
Ethnicity, *n* (%)					
Hispanic/Latino	81 (70)	82 (72)	82 (73)	82 (72)	327 (72)
Not Hispanic/Latino	34 (30)	32 (28)	31 (27)	32 (28)	129 (28)
Baseline patient-reported outcomes					
Rescue-free 24-h periods (SD), %	18.8 (34.20)	20.3 (35.65)	19.1 (33.73)	17.9 (33.60)	-
Symptom-free 24-h periods (SD), %	10.5 (22.50)	7.9 (19.98)	10.2 (22.35)	6.7 (19.30)	-
cACT score ≥20, *n* (%)	58 (50)	49 (43)	48 (42)	46 (40)	-

*cACT* childhood asthma control test, *ITT* intention-to-treat, *SD* standard deviation, *VI* vilanterol

**Table 2:** Lung function measures at screening and baseline (ITT population)

Lung function measures	Placebo	VI 6.25 μg	VI 12.5 μg	VI 25 μg	Total
	*N* = 115	*N* = 114	*N* = 113	*N* = 114	*N* = 456
Screening					
Pre-bronchodilator PEF (L/min)					
*n*	115	114	113	114	456
Mean	185.05	178.35	179.22	177.09	179.94
SD	59.30	55.67	60.57	55.68	57.74
Post-bronchodilator PEF (L/min)					
*n*	115	114	113	114	456
Mean	238.79	232.29	234.54	228.37	233.51
SD	67.41	69.80	69.79	72.29	69.71
Percentage of pre- to post-bronchodilator PEF (%)					
*n*	115	114	113	114	456
Mean	76.95	76.86	75.87	78.02	76.93
SD	9.18	9.57	10.90	8.17	9.50
Pre-bronchodilator FEV_1_ (L)					
*n*	91	91	97	102	381
Mean	1.405	1.370	1.374	1.361	1.377
SD	0.408	0.423	0.445	0.429	0.426
Post-bronchodilator FEV_1_ (L)					
*n*	99	98	96	103	396
Mean	1.700	1.702	1.699	1.680	1.695
SD	0.457	0.532	0.465	0.496	0.487
Pre-bronchodilator FEV_1_ (% predicted)^a^					
*n*	91	91	97	102	381
Mean	86.83	82.92	85.04	84.08	84.70
SD	19.83	17.00	17.34	16.77	17.73
Post-bronchodilator FEV_1_ (% predicted)^a^					
*n*	99	98	96	103	396
Mean	106.81	101.10	104.34	104.58	104.22
SD	20.94	18.47	16.68	19.97	19.15
Baseline					
Pre-bronchodilator PEF (L/min)					
*n*	115	114	113	114	456
Mean	186.27	185.08	181.24	179.39	183.00
SD	60.64	59.17	55.19	56.82	57.88
Percentage of pre- to post-bronchodilator PEF (%)					
*n*	115	114	113	114	456
Mean	77.77	79.54	77.58	79.13	78.51
SD	10.49	9.09	10.11	9.77	9.88
Pre-bronchodilator FEV_1_ (L)^b^					
*n*	96	91	97	103	387
Mean	1.367	1.408	1.361	1.365	1.374
SD	0.421	0.454	0.398	0.398	0.416
Pre-bronchodilator FEV_1_ (% predicted)^a,b^					
*n*	96	91	97	103	387
Mean	84.37	83.99	85.43	85.75	84.91
SD	19.35	16.99	17.66	18.38	18.08

*FEV*_*1*_ forced expiratory volume in one second, *ITT* intention-to-treat, *PEF* peak expiratory flow, *SD* standard deviation, *VI* vilanterol

^a^% predicted FEV_1_ data are posthoc analyses

^b^Three children had post-dose FEV_1_ at baseline and were not included in any FEV_1_ secondary endpoint analyses

### Primary endpoint

**Fig. 2:**
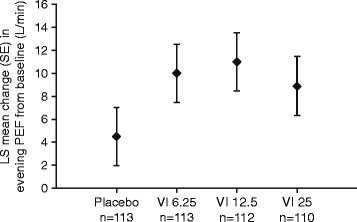
LS mean change from baseline in evening PEF averaged over Weeks 1 to 4 (ITT population). Abbreviations: *ITT*, intention-to-treat; *LS*, least squares; *PEF*, peak expiratory flow; *SE*, standard error, *VI*, vilanterol

The adjusted treatment differences in the mean change from baseline in daily pre-dose trough evening PEF versus placebo were 5.5 L/min, 6.4 L/min and 4.4 L/min, for the VI 6.25, VI 12.5 and VI 25 groups, respectively (Fig. 2 and Table 3). The adjusted treatment difference between the VI 25 and placebo groups was not statistically significant (*p* = 0.227). Therefore, in accordance with the step-down closed testing procedure, no statistical inference was made for the comparisons of VI 12.5 μg or VI 6.25 μg with placebo. The analysis of the primary endpoint using the PP population plus all sensitivity and supporting analyses using the ITT population supported the findings of the primary analysis. There was no apparent VI dose-ordering in the evening PEF treatment difference values from placebo.

**Table 3:** Secondary outcomes (ITT population)

	Placebo	VI 6.25 μg	VI 12.5 μg	VI 25 μg
	*N* = 115	*N* = 114	*N* = 113	*N* = 114
Primary endpoint				
Mean change from baseline in daily evening PEF Weeks 1–4 (L/min)				
*n*	113	113	112	110
LS mean	215.9	221.4	222.4	220.3
LS mean change (SE)	4.5 (2.53)	10.0 (2.53)	11.0 (2.54)	8.9 (2.56)
Diff. vs placebo		5.5	6.4	4.4
95 % CI		−1.6, 12.5	−0.6, 13.5	−2.7, 11.4
*p*-value		0.127	0.073	0.227
Secondary endpoints				
Change from baseline in trough FEV_1_ (L) at Week 4 (LOCF)				
*n*	85	83	86	86
LS mean	1.616	1.559	1.632	1.586
LS mean change (SE)	0.223 (0.029)	0.166 (0.029)	0.240 (0.029)	0.193 (0.029)
Diff. vs placebo		−0.057	0.017	−0.030
95 % CI		−0.138, 0.024	−0.063, 0.096	−0.110, 0.051
Change from baseline in percentage of rescue-free 24-h periods, Weeks 1–4				
*n*	113	113	112	110
LS mean change (SE)	14.4 (2.97)	12.2 (2.97)	15.8 (2.98)	23.1 (3.01)
Diff. vs placebo		−2.3	1.3	8.7
95 % CI		−10.5, 6.0	−6.9, 9.6	0.4, 17.0
Change from baseline in percentage of symptom-free 24-h periods, Weeks 1–4				
*n*	113	113	112	110
LS mean change (SE)	9.9 (2.65)	10.1 (2.65)	18.3 (2.66)	19.7 (2.69)
Diff. vs placebo		0.2	8.3	9.8
95 % CI		−7.2, 7.5	1.0, 15.7	2.3, 17.2
Change from baseline in morning PEF (L/min), Weeks 1–4				
*n*	114	113	112	110
LS mean	206.3	211.8	213.8	213.5
LS mean change (SE)	6.4 (2.42)	12.0 (2.43)	13.9 (2.44)	13.7 (2.46)
Diff. vs placebo		5.5	7.5	7.2
95 % CI		−1.2, 12.3	0.7, 14.2	0.4, 14.0
Change from Baseline in morning PEF (L/min), endpoint – Week 4 (LOCF)				
*n*	114	113	112	110
LS mean	207.2	213.1	216.9	214.2
LS mean change (SE)	7.4 (3.45)	13.3 (3.47)	17.0 (3.48)	14.4 (3.51)
Diff. vs placebo		5.9	9.7	7.0
95 % CI		−3.7, 15.6	0.0, 19.3	−2.7, 16.7
Change from Baseline in evening PEF (L/min), endpoint - Week 4 (LOCF)				
*n*	113	113	112	110
LS mean	217.3	220.8	225.1	222.5
LS mean change (SE)	5.9 (3.44)	9.4 (3.44)	13.7 (3.45)	11.1 (3.48)
Diff. vs placebo		3.5	7.8	5.2
95 % CI		−6.1, 13.1	−1.8, 17.4	−4.4, 14.9
Other endpoints				
Change from baseline in cACT score by baseline category at Week 4^a^				
Baseline cACT Score <20				
*n*	48	57	59	56
LS mean	19.5	19.8	20.2	20.9
LS mean change (SE)	3.5 (0.54)	3.8 (0.49)	4.2 (0.48)	4.9 (0.50)
Diff. vs placebo		0.3	0.7	1.4
95 % CI		−1.2, 1.7	−0.7, 2.1	−0.1, 2.9
Baseline cACT Score ≥20				
*n*	50	39	47	36
LS mean	22.9	21.5	21.9	22.5
LS mean change (SE)	0.8 (0.48)	−0.5 (0.53)	−0.2 (0.48)	0.5 (0.55)
Diff. vs placebo		−1.3	−1.0	−0.4
95 % CI		−2.8, 0.1	−2.4, 0.4	−1.8, 1.1

*cACT* childhood asthma control test, *CI* confidence interval, *FEV*_*1*_ forced expiratory volume in one second, *ITT* intention-to-treat, *LS* least squares, *PEF* peak expiratory flow, *SE* standard error, *VI* vilanterol

^a^cACT data are post-hoc analyses

### Secondary endpoints

Since the primary endpoint did not reach statistical significance, no inference was made for the secondary endpoints. A total of 384 (84 %) children completed a technically acceptable pre-bronchodilator FEV_1_ assessment at baseline. Of these, a total of 323 (71 %) children and 291 (64 %) children also completed an acceptable FEV_1_ assessment at Week 2 and Week 4, respectively. The analysis of change from baseline in trough FEV_1_ at Week 4 (LOCF) included 340 (75 %) children in the ITT population who provided technically acceptable FEV_1_ data both at baseline and at post-baseline. There was little difference in the change from baseline in trough FEV_1_ at Week 4 for each of the VI treatments compared with placebo (Table 3).

Over the treatment period, the greatest treatment difference in change from baseline in the percentage of rescue-free 24-h periods was observed in the VI 25 treatment group when compared with placebo (Table 3). This equated to an additional 0.6 rescue-free 24-h periods per week for children. For symptom-free days, the treatment differences observed between placebo and the VI 12.5 and VI 25 treatment groups, respectively, equate to an additional 0.6 and 0.7 symptom-free days per week. No difference in rescue-free or symptom-free days was found for the VI 6.25 group.

Across all secondary PEF endpoints, small but non-significant increases were seen with VI treatments compared with placebo. However, as with the primary endpoint, no dose–response was observed (Table 3).

Children whose asthma was controlled at baseline (cACT score ≥20) experienced only small improvements in cACT score following treatment with VI compared with placebo, whereas in children with a cACT score <20 at baseline, larger increases were observed (Table 3).

### Pharmacokinetics

In total, 341 children (PK population) provided 780 PK samples. There was a very high proportion of pre-dose samples with non-quantifiable levels of VI, especially in the two lower dose groups (91, 94 and 74 % for the VI 6.25, VI 12.5 and VI 25 populations, respectively). At 10−15 min post-dose, the proportion of samples below the LLQ was 48, 30 and 20 % for the VI 6.25, VI 12.5 and VI 25 groups, respectively. The concentration-time data for the current study were combined with data from previous studies conducted in children with asthma receiving either VI 25 μg alone [18] or in combination with FF [17]. The mean plasma concentrations of VI 10−15 min post-dose were dose ordered and as expected for the doses administered (17.27 pg/mL, 40.04 pg/mL and 90.47 pg/mL, after treatment with VI 6.25 μg, VI 12.5 μg and VI 25 μg, respectively).

### Safety

The incidence of AEs during treatment was higher in the VI groups (28–33 %) than in the placebo group (22 %), but there was no apparent dose-ordering (Table 4). The incidence of AEs considered to be drug-related by the investigator was 3 and 2 % in the VI 6.25 and VI 12.5 groups, respectively, with none reported in the placebo or VI 25 groups. The most frequently reported AE during the treatment period was nasopharyngitis. One child in each of the VI 6.25 and VI 25 groups experienced an AE leading to study withdrawal. The child in the VI 25 treatment group experienced an on-treatment non-fatal serious AE (SAE) of appendicitis and the child in the VI 6.25 treatment group experienced a viral respiratory tract infection. Neither AE was judged to be related to study treatment.

**Table 4:** Most frequent on-treatment AEs by preferred term (ITT population)

AE (preferred term), *n* (%)	Placebo	VI 6.25 μg	VI 12.5 μg	VI 25 μg
	*N* = 115	*N* = 114	*N* = 113	*N* = 114
Children with any AE	25 (22)	33 (29)	37 (33)	32 (28)
Children with most frequent events	15 (13)	27 (24)	26 (23)	18 (16)
Nasopharyngitis	8 (7)	8 (7)	10 (9)	9 (8)
Headache	4 (3)	6 (5)	2 (2)	2 (2)
Rhinitis	3 (3)	2 (2)	3 (3)	1 (<1)
Respiratory tract infection viral	1 (<1)	3 (3)	2 (2)	1 (<1)
Rhinitis allergic	0	2 (2)	3 (3)	2 (2)
Abdominal pain	1 (<1)	0	3 (3)	2 (2)
Bronchitis	2 (2)	3 (3)	1 (<1)	0
Pharyngitis	1 (<1)	3 (3)	0	2 (2)
Influenza	0	4 (4)	0	0
Ear pain	0	0	3 (3)	0

All treatments were administered on a constant background of open-label FP 100 μg twice daily. Table shows events that occurred in ≥3 % in any treatment group

*AE* adverse event, *ITT* intention-to-treat, *VI* vilanterol

Nine (2 %) children experienced asthma exacerbations during the treatment period (one child in the placebo group, three in the VI 6.25 group, one in the VI 12.5 group and four in the VI 25 group). None of these children were hospitalised but eight were withdrawn from the study as a result.

The mean changes from baseline in vital signs at Week 4 were small and similar between the placebo and VI treatment groups (least squares mean change in systolic BP: 0.1–1.1 mm Hg; diastolic BP: -0.1–0.4 mm Hg; pulse rate: -0.7–0.4 beats/min). Similarly, vital sign measurements at Week 2 and the maximum post-baseline changes showed only minor fluctuations from baseline. Abnormal ECG findings during the study were considered to be of potential clinical importance for 12 (3 %) children: two (2 %) children in the placebo group, six (5 %) children in the VI 6.25 μg group and two (2 %) children in each of the VI 12.5 and VI 25 groups. No AEs relating to ECG findings were reported during the study, and no children were withdrawn due to meeting the protocol-defined ECG stopping criteria.

## Discussion

In this study, we observed that once-daily inhaled VI added to a low dose of ICS in children with inadequately controlled asthma showed no statistically significant benefit in PEF measurements over placebo, which we believe also indicates a lack of clinical benefit for this outcome measure. The small numeric improvements over placebo in evening PEF following VI treatment were not statistically significant for the highest dose of 25 μg, and therefore no inferences were made for the lower doses investigated in this study. No apparent dose–response was observed. Therefore, the study was unable to demonstrate an incremental benefit in selected lung function measures with the addition of VI to open-label FP in this paediatric population.

We selected trough (evening) PEF as the primary endpoint because it was considered a more accessible measure of lung function than FEV_1_, as good quality spirometry may be difficult to perform in children [19]. However, PEF measurements have been reported to be variable [20, 21] and more so than FEV_1_ measurements [22]. Thus, PEF measurements may have introduced a level of under or over estimation of the treatment benefit. A previous study of the ICS/LABA combination FP/salmeterol in children ages 4−11 years, showed an increase in evening PEF compared with FP alone (mean change [standard error] from baseline over Weeks 1−12: 21.5 L/min [2.43] and 15.1 L/min [2.83], respectively) [23]. Furthermore, results of a Cochrane review comparing trials of 6–24 weeks duration in children found that a combination of LABA and ICS resulted in a significantly greater improvement in PEF compared with ICS monotherapy (morning PEF difference was 7.55 L/min and evening PEF was 5.5 L/min) [6]. The treatment differences reported in these analyses are in line with our results, although statistical significance was not achieved in this study.

The children in the current study had inadequately controlled asthma and a mean percentage of pre- to post-bronchodilator PEF of 76.93 %. Therefore, a benefit would be expected in these children from the addition of LABA to their existing ICS therapy. Baseline cACT scores suggested a high proportion of controlled children (40−50 % cACT score ≥20), which could provide a possible explanation for the lack of efficacy observed in this study. Another possible reason that no significant benefit in PEF was observed is that ICS provide good control of asthma in children, [24] thus reducing the potential for additional improvements in lung function. Of note, it is possible that children were more adherent to twice-daily ICS when in the trial compared with an unsupervised setting, as has been previously reported [25].

The mean pre- and post-bronchodilator FEV_1_ at screening was 84.7 and 104.2 %, respectively, suggesting that children recruited into this study had the potential for improvement in FEV_1_ measurements by use of a bronchodilator. Large increases from baseline in trough FEV_1_ at Week 4 (LOCF) were observed for both VI and placebo treatment groups, hence the small treatment differences versus placebo. Considering the small increases observed in morning and evening PEF, these increases in FEV_1_ may have been due to improved ability to perform spirometric measures over time, by both children and investigators, in addition to the benefits with ICS and improved adherence discussed above.

In the secondary efficacy endpoint analyses, an increase for rescue-free 24-h periods was observed in the VI 25 group compared with placebo. Additionally, notable increases in symptom-free 24-h periods were observed for both VI 12.5 and VI 25 groups. The increase in rescue-free 24-h periods observed in the VI 25 group and those in symptom-free 24-h periods in the VI 12.5 and VI 25 groups exceed the lower bounds of the published minimal important differences in adults [26]. This finding indirectly supports the importance of including more than just lung function measurements in the main analyses of studies in children. Indeed, a large long-term study in children with asthma aged 5–12 years found that ICS treatment had no effect on lung function but resulted in improvements in airway responsiveness and health outcomes over the 4–6-year treatment period [27].

At the two lower doses of VI, the majority of PK samples were below quantifiable levels. The mean plasma concentration of VI 10−15 min post-dose was dose-ordered and systemic exposure observed for VI 25 μg was similar to that observed in a Phase IIa study of FF/VI 100/25 μg in children [18]. As only one post-dose blood sample was collected, conclusions on maximum plasma concentration (C_max_) and time to maximum plasma concentration (t_max_) cannot be drawn from this study. However, studies in adults suggest that VI 25 μg is rapidly absorbed and eliminated from the systemic circulation [28, 29] and in children VI 25 μg has been shown to reach a C_max_ shortly after dosing (t_max_ of 12 min) [18].

The incidence of overall AEs was similar across the VI treatment groups and slightly higher than in the placebo group, although there was no dose–response. The most common on-treatment AEs were those commonly observed in paediatric populations with asthma. The single SAE reported was not considered to be related to study treatment and there were no asthma-related hospitalisations or fatalities reported. The low number of exacerbations in each treatment group prevents meaningful comparisons between treatments.

Potential limitations of this study were the difficulty in recruiting children with poorly-controlled asthma on a background of ICS, and the variability of PEF measurements which may have further masked any treatment differences. The difficulty in obtaining FEV_1_ in young children means that using this as a primary endpoint requires an intensive programme to support coaching good quality spirometry from all children at all visits. The high number of study assessments was also a barrier to recruitment, and we recommend that study assessments are kept to a minimum for studies in children with asthma. Moreover, additional inclusion criteria based on cACT score at baseline, reflecting inadequately controlled asthma, could be used to ensure recruitment of a more clinically relevant population.

## Conclusion

In summary, we found that the three doses of VI investigated in this study (6.25, 12.5 and 25 μg) did not significantly improve the change from baseline in daily pre-dose trough evening PEF in children ages 5−11 years and no dose–response was observed in change from baseline in evening PEF. Notable improvements over placebo were seen for VI 25 treatment in the percentage of rescue-free and symptom-free 24-h periods. All treatments were well tolerated and no new safety concerns were identified during the study.

## Abbreviations

AE: adverse event
ANCOVA: analysis of covariance
BP: blood pressure
cACT: childhood asthma control test
CI: confidence interval
C_max_: maximum plasma concentration
ECG: electrocardiogram
FEV_1_: forced expiratory volume in one second
FF: fluticasone furoate
FP: fluticasone propionate
ICS: inhaled corticosteroids
ITT: intention-to-treat
LABA: long-acting beta-2 agonist
LLQ: lower limit of quantification
LOCF: last observation carried forward
LS: least squares
MMRM: mixed modelling repeated measures
PEF: peak expiratory flow
PK: pharmacokinetic
PP: per protocol
SABA: short-acting inhaled beta-agonist
SAE: serious adverse event
SD: standard deviation
SE: standard error
t_max_: time to maximum plasma concentration
VI: vilanterol
